# Effects of myofascial release on frequency of joint bleedings, joint status, and joint pain in patients with hemophilic elbow arthropathy: A randomized, single-blind clinical trial

**DOI:** 10.1097/MD.0000000000026025

**Published:** 2021-05-21

**Authors:** Rubén Cuesta-Barriuso, Raúl Pérez-Llanes, Elena Donoso-Úbeda, José Antonio López-Pina, Javier Meroño-Gallut

**Affiliations:** a Department of Physiotherapy, Faculty of Sport Sciences, European University of Madrid; b Royal Victoria Eugenia Foundation; c Fishemo CEE, Spanish Federation of Hemophilia, Madrid; d Department of Physiotherapy, Catholic University San Antonio-UCAM; e Optimus Osteopathy and Physiotherapy Clinic; f Department of Basic Phycology and Methodology, University of Murcia; g Tú. Bienestar 360°, Physiotherapy and Medical Center, San Javier-Murcia, Spain.

**Keywords:** elbow, hemophilic arthropathy, myofascial release, physical therapy, randomized clinical trial

## Abstract

**Background:**

Chronic joint injury of the elbow joint is common in patients with hemophilia. Myofascial release is used for the management of pain and functionality in patients with chronic restrictions.

**Objective:**

To evaluate the effectiveness of myofascial release in patients with hemophilic elbow arthropathy.

**Methods:**

Sixty-nine patients with hemophilia took part in this randomized controlled trial. They were recruited from 10 hemophilia patient Associations. They were randomly allocated to experimental (n = 35) or control group (n = 34). The intervention consisted of three 50-min sessions of fascial therapy over a 3-week period. The intervention included 11 bilaterally administered maneuvers in both upper limbs (from shoulder girdle to forearm). The study variables were frequency of elbow bleeding (self-report), joint status (Hemophilia Joint Health Score), and joint pain (visual analog scale) at baseline, after the intervention, and at the 3-month follow-up.

**Results:**

There were significant changes (*P* < .001) in the repeated measures factor in the frequency of hemarthrosis (*F* = 20.64), joint status (*F* = 31.45), and perceived joint pain (*F* = 30.08). We found group interaction with the (*P* < .001) in the frequency of hemarthrosis (*F* = 21.57), joint status (*F* = 99.98), and perceived joint pain (*F* = 44.26). There were changes (*P* < .01) in the pairwise comparison analysis between the pretreatment assessment and the posttreatment and follow-up assessments.

**Conclusions:**

Myofascial release decreases frequency of elbow bleedings, and improved joint status and perception of elbow pain in patients with hemophilic elbow arthropathy. Myofascial release may be recommended to improve joint status and joint pain in patients with hemophilic elbow arthropathy.

## 1 Introduction

Hemophilia is a X-linked congenital coagulopathy characterized by a deficit of clotting factors: hemophilia A (clotting factor VIII [FVIII]) or hemophilia B (clotting factor IX [FIX]).^[1]^ The main clinical symptoms are joint bleeding (hemarthrosis).^[2]^ The recurrence of bleeding episodes leads to the development of synovial hypertrophy and bone and cartilage destruction.^[3]^ Consequences for the joint as a result of a succession of repeated hemarthrosis events in a single joint include hemophilic arthropathy.^[4]^

The only treatment that has shown to be effective in the prevention of the development of hemophilic arthropathy is the prophylactic treatment.^[5]^ The elbow, along with the ankle and knee, are the joints most affected by arthropathy in patients with hemophilia.^[6]^

Hemophilic arthropathy in the elbow joint is characterized by hypertrophy of the head of radius, together with the destruction of the humeroulnar joint, which may cause a severe restriction of forearm mobility, debilitating and chronic pain, and even joint ankylosis.^[7]^ However, research on the safety and efficacy of physiotherapy in elbow arthropathy is limited.^[8]^ Some interventions based on swimming^[9]^ and endurance training^[10]^ have shown an improvement in the perception of joint pain, and a reduced frequency of elbow bleeds, respectively.

Fascial therapy is indicated in the treatment of chronic degenerative lesions. The myofascial release technique is a manual physiotherapy technique that applies the principles of biomechanical loading of the soft tissue and the modifications of the neural reflexes, through stimulation of the mechanoreceptors in the fascia with the purpose of releasing fascial restrictions and restoring healthy tissue.^[11,12]^ The aim is to manipulate the fascial complex over long periods of time, to improve tissue mobility, pain, and joint, muscular, and proprioceptive functionality. Fascial therapy in patients with hemophilia is contraindicated due to the risk of developing muscle or joint hemorrhages as a result of superficial, deep, or telescopic techniques typically applied with this therapy. Although pilot and cohort studies^[13,14]^ have demonstrated the effectiveness of this technique in patients with hemophilic ankle arthropathy, no studies have evaluated the safety and efficacy in the approach to elbow arthropathy. The use of these techniques, following definite protocols and applied by professionals trained in the management of patients with hemophilia, appears to be safe.

We hypothesized that an intervention protocol combining manual therapy in patients with hemophilic elbow arthropathy would improve frequency of clinical hemarthrosis, joint health, and perceived pain, as compared to a sham intervention. The objective of this study was to examine the effectiveness of manual therapy through myofascial release regarding the frequency of joint bleedings in patients with hemophilic elbow arthropathy, and to explore the myofascial release-induced changes in joint status and elbow joint pain.

## 2 Material and methods

### 2.1 Study design and ethical issues

A single-blind randomized controlled trial with a 3-month follow-up period was approved by the Research Ethics Committee of the University of Murcia (ID: 1505/2017) and registered in the clinical trial.gov (ClinicalTrials.gov NCT03009591). All participants provided written informed consent, while all procedures were conducted in accordance with the principles of the Declaration of Helsinki.

The sample size was established taking into account the frequency of joint bleedings for the evaluation of safety as a primary outcome, and was carried out on the basis of the following assumptions: significance level (α) = 0.05, type 2 error (β) = 0.2; with an 90% test power. For sample calculation, G∗Power 3.1.9.4 was used based on an effect size of 0.8 (large effect size as Cohen) and the Bonferroni correction for multiple comparisons. Twenty-eight patients per group thus determined the calculated sample size. Considering a 20% drop-out, the total sample was made up of a minimum of 67 patients.

An administrative assistant, blinded to the objectives of the study, carried out patient allocation to each of the groups. Patients were randomly allocated into groups receiving myofascial release treatment (n = 35) and no intervention (n = 34), according to an opaque envelope system.

### 2.2 Inclusion/exclusion criteria and patients recruited

Patients with hemophilic elbow arthropathy were recruited from 10 hemophilia patient associations around Spain (Bilbao, Burgos, Galicia, Madrid, Málaga, Murcia, Salamanca, Valencia, Valladolid, and Zaragoza) from March 2018 to February 2019. The inclusion criteria to participate in the study were: patients diagnosed with hemophilia A and B; type of hemophilia severity (severe or moderate); of legal age; and medical diagnosis of hemophilic elbow arthropathy (more than 3 points on the Hemophilia Joint Health Score).

Similarly, patients excluded from the study were those: without walking ability; with neurological or cognitive disorders that prevented understanding the questionnaires and physical tests; and failing to sign the informed consent document. This study did not exclude patients with hemophilia based on their type of medical treatment, including both patients on demand and prophylactic treatments. Patients on prophylactic treatment continued to receive the dose of FVIII/FIX concentrates prescribed in accordance with the orders given by their hematologist. Similarly, patients who had developed antibodies to FVIII/FIX (inhibitors) but currently had no inhibitor titers were included.

### 2.3 Intervention

The experimental group received 3 interventions of myofascial release. Each session lasted 50 minutes, with 3 physiotherapy sessions over a 3-week period. The manual therapy intervention was carried out by a physiotherapist with more than 10 years of experience in fascial therapy and hemophilia. The treatment program implemented^[15]^ included 11 maneuvers, including the administration of 7 superficial maneuvers, and 4 deep maneuvers. The intervention included manual therapy techniques on both lower limbs, including forearm, elbow, arm, and shoulder girdle.

The 7 superficial techniques were:

(1) longitudinal surface sliding maneuver on the surface fascia in the anterior region of the arm and forearm;
(2) transverse sliding maneuver of the flexor muscles of the wrist and fingers;
(3) transverse sliding maneuver on the brachial biceps;
(4) longitudinal surface sliding maneuver on the surface fascia in the posterior region of the arm;
(5) transverse sliding maneuver on the tendon of the brachial triceps;
(6) transverse sliding maneuver for the pectoralis major muscle; and
(7) transverse sliding maneuver for the posterior axillary region (complex dorsi, subscapularis, teres minor and major, and pectoralis major muscle).

All surface techniques were applied using 3 strokes of 15 slides, 1 minute per technique approximately.

**Figure 1:**
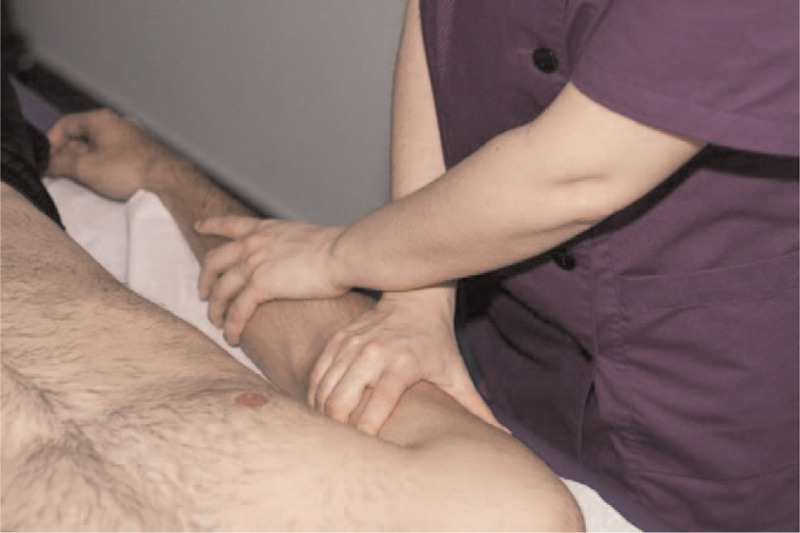
Induction maneuver: hands crossed on the brachial region and forearm.

**Figure 2:**
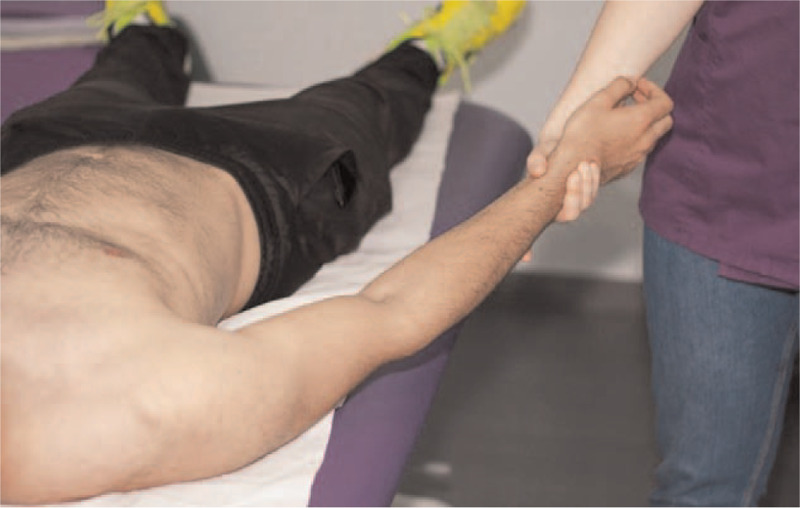
Telescopic maneuver of the upper limb.

**Figure 3:**
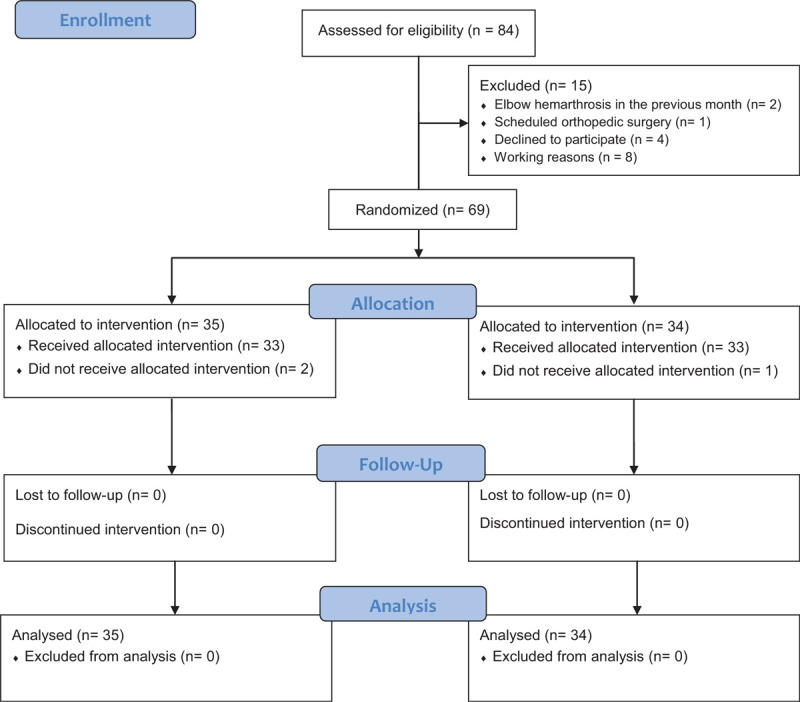
Flow diagram of patients with hemophilia in the randomized controlled trial.

The 4 deep techniques applied were:

(1) induction maneuver of the fascia of the posterior axillary fold;
(2) induction maneuver: hands crossed on the brachial region and forearm (Fig. 1);
(3) transverse plane maneuver for the cervicothoracic region; and
(4) telescopic maneuver of the upper limb (Fig. 2).

The duration of these deep techniques was 3 to 5 minutes in the first 2 and 5 minutes in the remaining 2.

The subjects included in the control group received no intervention and continued with their usual routine of activity and physical exercise, as well as with their prescribed treatment with FVIII/FIX concentrates. After the follow-up period, the patients in the control group received the same intervention and under the same conditions as the patients in the experimental group.

### 2.4 Outcome measures

Three evaluations were carried out in this study: before intervention (T0), following intervention (T1), and after a 3-month follow-up period (T2). The evaluations were carried out on the premises of the hemophilia associations included in the study. All elbow joints with hemophilic arthropathy were evaluated, regardless of dominance. The same physiotherapist with more than 15 years of experience in the treatment of patients with hemophilia, was responsible for conducting the assessments following the same guidelines and under the same conditions, blinded regarding the allocation of patients to study groups.

The primary outcome was the frequency of joint bleedings developed by patients with hemophilia during the treatment and intervention period. This frequency of hemarthrosis was determined by a self-report provided at the beginning of the study. This self-report was given to the patient during the various evaluations. Similarly, within 48 hours following the intervention, telephone follow-up was carried out to establish the presence of hemorrhagic complications during that period, as a result of the intervention.

The perception of elbow joint pain was evaluated with the visual analog scale. This measuring instrument is 10-cm line, with an end point of 0 indicating “no pain” and 10 representing “the worst pain.” Patients were asked to make a mark on the line to represent their intensity of pain in the elbow joint, assessing the level of intensity of pain by measuring the distance from the “no pain” end to the mark made by the patient. The reliability and validity of this scale in the assessment of pain has been demonstrated^[16]^ and has been widely used in the assessment of pain perception in patients with hemophilic arthropathy.^[17,18]^

The joint state was evaluated using version 2.1. of the Hemophilia Joint Health Score^[19]^ using the validated Spanish version of this specific hemophilia questionnaire for the assessment of the joint health. This scale that includes 8 items (joint swelling, duration of swelling, muscle atrophy, strength, crepitus on motion, flexion and extension loss, and pain), scores from 0 to 20 points per joint (the higher the score, the greater the degree of joint deterioration). It is applied to the joints of the knees, ankles, and elbows, as well as a global gait score.^[20]^

For the measurements, a pilot was run to determine the interrater reliability. The physiotherapist who performed the assessments and another expert physiotherapist, took part in this pilot study of 7 patients with hemophilia with elbow arthropathy. The frequency of elbow hemarthrosis, joint status, and elbow pain were measured.

At the beginning of the study, the main anthropometric independent variables (weight, height, and body mass index), clinical variables (type and severity of hemophilia, type of treatment, and history of inhibitors), and sociodemographic variables (age, dominance) were evaluated.

### 2.5 Statistical analysis

The statistical analysis was carried out with version 19.0 of the statistical package SPSS for Windows (IBM Company, Armonk, NY).

The intraclass correlation coefficient (ICC) was used to evaluate the interrater reliability. This intraexaminer reliability was obtained using the measurements performed by the examiner in this trial. Clinical and anthropometric characteristics were compared between both groups using Fisher exact tests for categorical variables and Kolmogorov–Smirnov for continuous variables.

The minimum detectable change (MDC) was calculated. For its calculation, the standard error of measurement (SEM) was estimated. SEM was calculated using the following formula: SEM = SD_pre_∗√1-*R*, with *R* being the reliability coefficient.^[21]^ As a measure of reliability we use Cronbach alpha coefficient.^[22]^ Based on the SEM, the MDC was obtained (MDC = *Z*-score ∗ √2∗SEM). The confidence level was set at 95% (*Z*-score = 1.96).^[23]^ An analysis of variance of repeated measures was carried out to compare the experimental and control groups at the 3 assessment times: baseline (T0), posttreatment (T1), and follow-up (T2). The results of the *F* test depend on whether the Mauchly spherical test was significant or not. If significant, the Greenhouse–Geisser correction was used. Bonferroni correction has been applied to control the error rate of the significance level. When the interaction was significant, pairwise comparison tests were performed on the group. The partial eta-squared value was calculated as an indicator of effect size (classified as small 0.01, medium 0.06, and large 0.14).^[24]^

When performing the statistical analysis, all subjects were considered based on an intention-to-treat analysis. The chosen level of significance was α = 0.05.

## 3 Results

Sixty-nine patients with hemophilic elbow arthropathy were included in the study. During the intervention period, 2 patients in the experimental group and 1 in the control group dropped out of the study for work reasons. During the follow-up period, none of the subjects left the study and were evaluated 3 months after the end of treatment. Thus, 66 patients with hemophilic elbow arthropathy completed the study (Fig. 3). Most of the patients (89.8%) had bilateral elbow arthropathy.

Interrater reliability for the physical variables was high (*P* < .001), with significant interrater correlations for frequency of elbow hemarthrosis (ICC = 1.00), joint status (ICC = 0.89), and elbow pain (ICC = 1.00).

**Figure 4:**
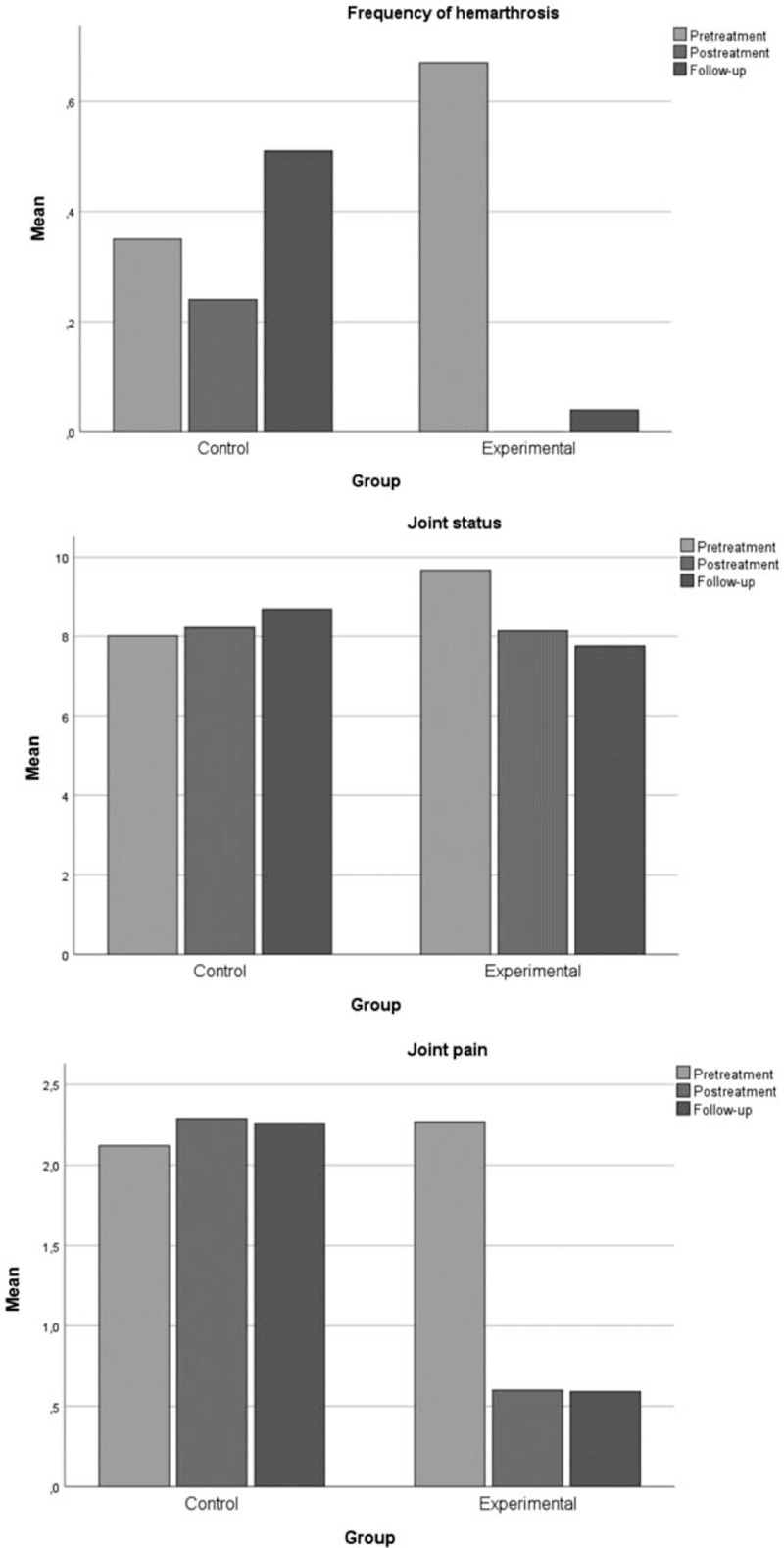
Changes in the study variables in each assessment, depending on the group.

Both groups were comparable in clinical characteristics. At baseline we found only differences between the 2 groups in the variables of hemophilia severity and right-hand dominance (Table 1). Table 2 shows the statistics regarding central tendency and dispersion. Figure 4 shows the changes in the study variables in each assessment depending on the group.

**Table 1:** Descriptive characteristics of all patients (n = 69) at baseline and in each group of the study.

	Experimental group		Control group		
Variables	M (SD)	Range	M (SD)	Range	Sig.
Age of patient (yr)	42.14 (10.41)	21–63	40.53 (9.98)	22–64	0.35^†^
Weight of patient (kg)	79.29 (14.10)	48–117	81.79 (12.27)	60–116	0.05^†^
Height of patient (cm)	172.43 (7.22)	158–187	174.71 (6.70)	165–191	0.26^†^
Body mass index (kg/m^2^)	26.61 (3.93)	19.42–35.32	26.77 (3.67)	20.28–39.67	0.79^†^

Clinical variables	n	n	Sig.
Type of hemophilia (A/B)	28/7	32/2	0.20^‡^
Severity (severe/moderate)^∗^	29/6	32/2	0.02^‡^
Treatment (prophylaxis/on demand)	25/10	28/6	0.28^‡^
Background of inhibitors (no/yes)	28/7	30/4	0.35^‡^
Dominance (right-hand/left-hand)^∗^	31/4	34/0	0.04^‡^

**Table 2:** Means (and standard deviations) of frequency of joint bleeding, joint status, and joint pain of the elbows with hemophilic arthropathy evaluated in the different assessments.

Variable	Group	T0 M (SD)	T1 M (SD)	T2 M (SD)	MDC
Frequency of hemarthrosis (number)	Experimental	0.67 (1.10)	0.00 (0.00)	0.04 (0.20)	1.99
	Control	0.35 (0.68)	0.24 (0.55)	0.51 (0.98)	
Joint status (HJHS; range: 0–20)	Experimental	9.67 (3.87)	8.14 (3.75)	7.76 (3.79)	1.90
	Control	8.01 (4.20)	8.22 (4.29)	8.69 (4.38)	
Joint pain (VAS; range: 0–10)	Experimental	2.27 (2.21)	0.60 (1.40)	0.59 (1.33)	2.31
	Control	2.12 (2.54)	2.29 (2.53)	2.26 (2.47)	

A decrease was noted in the frequency of elbow joint bleedings that varied depending on the time point of the evaluation and between the 2 groups was the same all the 3 times it was analyzed. Differences were noted for the joint status variable depending on the time of assessment, and between the 2 groups in the 3 evaluations. Perception of elbow pain changed depending on the time of assessment, and the difference between the 2 groups was not the same at the 3 times analyzed, being higher in the experimental group (Table 3).

**Table 3:** Intrasubject and intersubject effects test results in each one of the dependent variables of the study, among the study groups.

	Sphericity test		Intrasubject effect			Intersubject effect		
Variable	*W*	Sig.	*F*	Sig.	*η*^2^_*p*_	*F*	Sig.	*η*^2^_*p*_
Frequency of hemarthrosis (number)^∗^	0.56	.00^‡^	20.64	.00^‡^	0.13	21.57	.00^‡^	0.13
Joint status (HJHS; range: 0–20)^∗^	0.95	.04^†^	31.45	.00^‡^	0.18	99.98	.00^‡^	0.42
Joint pain (0–10)^∗^	0.48	.00^‡^	30.08	.00^‡^	0.18	44.26	.00^‡^	0.24

Pairwise comparison analysis showed an improvement in terms of frequency of joint bleeding, elbow joint health, and joint pain during the study period. There was a significant improvement after the intervention (T0–T1) and at follow-up compared with the baseline value (T0–T2) for all the dependent variables evaluated (Table 4).

**Table 4:** Pairwise comparison analysis between the assessments carried out.

	T0–T1		T0–T2	
Variable	MD (CI)	Sig.	MD (CI)	Sig.
Frequency of hemarthrosis (number)	0.39 (0.21; 0.57)	.00^†^	0.23 (0.06; 0.40)	.00^∗^
Joint status (HJHS; range: 0–20)	0.66 (0.45; 0.86)	.00^†^	0.61 (0.37; 0.86)	.00^†^
Joint pain (VAS; range: 0–10)	0.74 (0.43; 1.06)	.00^†^	0.76 (0.44; 1.09)	.00^†^

## 4 Discussion

The data obtained in the present investigation showed that physiotherapy intervention through myofascial release technique is safe and effective for the treatment of patients with patients with hemophilia and elbow arthropathy.

The implementation of this physiotherapy program has caused no events of elbow hemarthrosis during the intervention period, significantly decreasing the frequency of bleeding throughout the follow-up period. One of the main risks in the administration of physiotherapeutic treatment in patients with hemophilia is the possible development of hemarthrosis, although most manual techniques have shown to be safe and effective in reducing the frequency of bleeding.^[8,14,25]^ In addition to the safety of myofascial release reported in this study, improvements in pain and joint health of the elbow joint are noted. These findings have also been observed in connection with sports such as swimming^[9]^ for which a 10-month program showed improvements in joint condition and pain in patients with hemophilic elbow arthropathy.

Controlling the perception of pain combined with the administration of a prophylactic treatment are key aspects to reduce the frequency of bleeding and pain, thus improving joint function.^[26]^ Chronic pain, characteristic of hemophilic arthropathy, significantly affects the performance of daily life activities and the perception of quality of life in patients with hemophilia.^[27]^ Following a myofascial release intervention, we observed a significant improvement in the perception of pain in the elbow joint. The improvements were maintained even after the follow-up period. This finding is a relevant aspect considering the severe joint damage of the joints treated at the beginning of the study. Likewise, it should be considered that patients on demand treatment, without prophylaxis or hemostatic coverage with factor VIII / IX, were included. These results are consistent with the improvement achieved through the use of manual orthopedic therapy with joint traction to reduce pain.^[8]^ However, the results obtained with myofascial release became apparent after only 3 treatment sessions.

When arthropathy is established, the elbow extension movement is restricted, following the antalgic pattern of the acute hemarthrosis process, characterized by antalgic flexion (accompanied by functional impotence, inhibitory reflex of the muscle, and extremely intense pain). Both the acute and chronic process lead to the shortening of the periarticular structures, mainly soft tissue and flexor muscles. The improvement in joint condition after the intervention period, also maintained after the 3-month follow-up, may suggest the effectiveness of this intervention. In fact, the administration of myofascial release techniques has already shown to effectively reduce pain and disability, and to improve flexibility and functionality in every-day activities.^[28]^ The alteration of fascial pliability could be a source of body misalignment, potentially leading to poor muscular biomechanics, altered structural alignment, and decreased strength and motor coordination.^[29]^ This paper reports the clinical improvement of the joint condition in patients with hemophilic elbow arthropathy, and thus improving functionality and performance in the daily activities of patients.

This protocol of myofascial release techniques involves performing a slight biomechanical load on the soft tissue and, through the stimulation of the mechanoreceptors in the fascia, releasing the fascial constraints and restoring healthy tissue.^[12]^ This stimulation aims to reduce fascial system restrictions by promoting the reorientation of collagen fibers^[11]^ may promote improvements in terms of pain and functionality, and thus in the perceived disability in patients with hemophilic arthropathy.

Consideration of the effect size in the context of the hypothesis test is a way to control both the value of α (probability of making a mistake of type I), and the value of β (probability of making a type II error). Our study showed that statistically significant differences with large effect sizes were obtained posttreatment and at 3-month follow-up in patients treated with myofascial release techniques. This is highly relevant considering the small, but statistically significant changes reported in the literature following various treatments. A training program which lasted twelve months included physical exercise routines and swimming practices showed decrease of elbow pain.^[9]^ A recent study through joint traction^[8]^ found a significant improvement of frequency of joint bleedings of 0–1 and elbow joint pain of 0.5–0 after treatment period. Our effect size reported is important as they give clinical professional the means to objectively interpret improvements seen after myofascial release treatment between assessment sessions in patients with hemophilic elbow arthropathy.

The MDC is defined as the smallest amount of change outside the measurement error in the score of an instrument used to measure a symptom. Our results provided changes slightly lower than this value for the variables frequency of hemarthrosis (0.67 vs 1.99), joint status (1.53 vs 1.90), and joint pain (1.67 vs 2.31). These results should be interpreted taking into account the sample size and advanced joint damage of the patients included in the study.

The safe administration of this physiotherapy technique provides new therapeutic possibilities in the approach to hemophilic elbow arthropathy. The short duration of the intervention involved for the administered protocol, the scarce means needed to carry it out and the perception of improvement in the subjects since the very first session can favor its implementation as a treatment tool.

In this study with hemophilia patients, the requirements indicated in a recent publication^[30]^ have been met. Randomized trials on myofascial release include a-priori calculated sample sizes, limiting the sources of bias, follow-up time points beyond 24 hours, appropriate levels of significance, and appropriate registration. Randomized clinical trials should present between group differences and not report from reporting within group differences as evidence of effectiveness.

Some limitations of this study must be reported. An experimental control group with another intervention was not included as it was decided to compare the association of the protocol of myofascial release techniques with a group of patients who do not usually receive physiotherapy treatment. The measurement of the functionality or quality of life could have offered more complete results on the effectiveness of the manual therapy in patients with hemophilic elbow arthropathy.

## 5 Conclusion

The results of this study demonstrated the safety of a physiotherapy protocol using myofascial release in patients with hemophilic elbow arthropathy. This study provides evidence for the use of manual therapy through myofascial release to decrease frequency of hemarthrosis and improve joint status and elbow pain in patients with hemophilic elbow arthropathy. These improvements are maintained for a 3-month period. Thus, manual therapy treatment through myofascial release may be the treatment of choice in patients with hemophilic elbow arthropathy reporting high levels of joint deterioration and joint pain.

## Acknowledgments

The authors are especially grateful to the Spanish Federation of Haemophilia, and Galician Malaga, Vizcaya, Community of Valencia, Aragón-La Rioja, Valladolid-Palencia, Murcia Burgos, and Salamanca Associations of Hemophilia for their help in recruiting the sample.

## Author contributions

**Conceptualization:** Rubén Cuesta-Barriuso, Raúl Pérez-Llanes, José Antonio López-Pina, Javier Meroño-Gallut.

**Data curation:** Raúl Pérez-Llanes, Elena Donoso-Úbeda, José Antonio López-Pina, Javier Meroño-Gallut.

**Formal analysis:** Rubén Cuesta-Barriuso, José Antonio López-Pina.

**Funding acquisition:** Rubén Cuesta-Barriuso.

**Investigation:** Rubén Cuesta-Barriuso, Javier Meroño-Gallut.

**Methodology:** Rubén Cuesta-Barriuso, Raúl Pérez-Llanes, Elena Donoso-Úbeda, José Antonio López-Pina, Javier Meroño-Gallut.

**Project administration:** Rubén Cuesta-Barriuso.

**Resources:** Rubén Cuesta-Barriuso, Raúl Pérez-Llanes, Elena Donoso-Úbeda.

**Software:** Rubén Cuesta-Barriuso, José Antonio López-Pina, Javier Meroño-Gallut.

**Supervision:** Raúl Pérez-Llanes, Elena Donoso-Úbeda, José Antonio López-Pina, Javier Meroño-Gallut.

**Validation:** Rubén Cuesta-Barriuso, Javier Meroño-Gallut.

**Visualization:** Elena Donoso-Úbeda, José Antonio López-Pina.

**Writing – original draft:** Rubén Cuesta-Barriuso, José Antonio López-Pina, Javier Meroño-Gallut.

**Writing – review & editing:** Rubén Cuesta-Barriuso, Javier Meroño-Gallut.
